# Stereotype confirmation concerns predict dropout from cognitive behavioral therapy for social anxiety disorder

**DOI:** 10.1186/s12888-014-0233-8

**Published:** 2014-08-19

**Authors:** Suzanne Johnson, Matthew Price, Natasha Mehta, Page L Anderson

**Affiliations:** Georgia State University, Urban Life Building, 140 Decatur Street, Atlanta, GA 30308 USA; University of Vermont, Burlington, USA

**Keywords:** Social anxiety, Dropout, Stereotypes, Virtual reality exposure, Cognitive behavioral therapy

## Abstract

**Background:**

There are high attrition rates observed in efficacy studies for social anxiety disorder, and research has not identified consistent nor theoretically meaningful predictors of dropout. Pre-treatment symptom severity and demographic factors, such as age and gender, are sometimes predictive of dropout. The current study examines a theoretically meaningful predictor of attrition based on experiences associated with social group membership rather than differences between social group categories--fear of confirming stereotypes.

**Methods:**

This is a secondary data analysis of a randomized controlled trial comparing two cognitive behavioral treatments for social anxiety disorder: virtual reality exposure therapy and exposure group therapy. Participants (N = 74) with a primary diagnosis of social anxiety disorder who were eligible to participate in the parent study and who self-identified as either “African American” (n = 31) or “Caucasian” (n = 43) completed standardized self-report measures of stereotype confirmation concerns (SCC) and social anxiety symptoms as part of a pre-treatment assessment battery.

**Results:**

Hierarchical logistic regression showed that greater stereotype confirmation concerns were associated with higher dropout from therapy--race, age, gender, and pre-treatment symptom severity were not. Group treatment also was associated with higher dropout.

**Conclusions:**

These findings urge further research on theoretically meaningful predictors of attrition and highlight the importance of addressing cultural variables, such as the experience of stereotype confirmation concerns, during treatment of social anxiety to minimize dropout from therapy.

## Background

Attrition is an important issue in the practice and research of psychotherapy. People who suffer from treatable disorders may not benefit from evidenced-based therapies if they drop out of treatment prematurely. For example, considerable evidence shows that cognitive behavioral and exposure-based interventions are effective when used to treat social anxiety disorder [1,2]; many participants, however, leave treatment prematurely [3]. Unfortunately, predictors of dropout from treatment for social anxiety disorder are poorly understood.

A recent review of 16 randomized controlled trials found no consistent predictors of dropout [4]. Severity of social anxiety symptoms predicted dropout in two efficacy trials, but with different effects. The first indicated a positive relation between pre-treatment levels of social anxiety and dropout [5], whereas the second showed a negative relation [6]. Many other studies have not found symptom severity to be a predictor of attrition [7,8]. Therefore, research does not consistently support the idea that participants with social anxiety disorder are discontinuing treatment because of the intensity of their social anxiety symptoms when confronted with new social environments.

Demographic factors also have been examined as predictors of attrition from treatment for social anxiety disorder. Younger participants and women were more likely to drop out in two studies [9,10], but age and gender were not significant predictors of attrition in other studies [7,8]. Furthermore, scholars have criticized the use of demographic factors (including gender, age, and race) as predictors in the absence of other contextual data, as it provides limited information for interpretation [11]. Another approach to understanding the impact of social identities on attrition is to examine the experiences associated with social group membership rather than differences between social group categories. To that end, the current study examined stereotype confirmation concerns as a predictor of attrition.

Stereotypes have long been known to impact mental health and behavior [12]. People with high stereotype confirmation concerns “chronically experience uncertainty and apprehension about appearing to confirm as self-characteristic a stereotype about a group to which they belong” [13] p. 1778. Stereotype confirmation concerns can apply to any of the social groups with which individuals identify (e.g., groups based on gender, racial, sexual, and/or religious identity). All racial groups show a positive relation between stereotype confirmation concerns, stress and negative mood [13]. However, African Americans report the highest levels of stereotype confirmation concerns relative to Latinos, Asians, and Caucasians [13].

Stereotype confirmation concerns (SCC) may be useful for understanding the treatment behavior of people with social anxiety. The negative, distorted image of oneself that is characteristic of socially anxious individuals [14] may include the stereotypes about the social groups to which they belong. A person with social anxiety disorder could fear acting in a way that confirms stereotypes because it may lead to her rejection and also perpetuate negative stereotypes about her social group. Only two case studies have explicitly discussed the impact of stereotypes or fears of confirming them on treatment for social anxiety [15,16]. Johnson [16] argued that the expectation of being judged according to negative racial stereotypes is a prominent fear among African American college students and presented a case study of an African American female whose treatment involved identification of race-related triggers of her social anxiety. Similarly, in a case study of an African American woman with social anxiety disorder, Fink, Turner, & Beidel [15] found that the fear of being judged according to stereotypes was a central feature of social fears; racially relevant interpersonal contexts served as antecedents for fear of negative evaluation, and directly addressing these fears during exposure therapy was effective. These studies highlight that racial stereotypes and the fear of being evaluated according to them can be important triggers [of social anxiety] and [may be] beneficial when incorporated into treatment of social anxiety disorder. Future research is necessary to generalize the findings of these case studies by examining the impact of stereotypes confirmation concerns on those with social anxiety disorder and on their treatment.

No research to date has examined whether or not stereotype confirmation concerns are related to attrition from treatment. People with high stereotype confirmation concerns may be at greater risk to drop out of therapy for fear of confirming therapists’ stereotypes about the client’s social groups. If participating in group therapy, the gold standard treatment for social anxiety disorder, a person may also have concerns about confirming stereotypes of other group members.

We investigate the relation between SCC and dropout from cognitive behavioral therapy for social anxiety in two different formats (individual and group) among a sample of participants who self-identify as either “African American” or “Caucasian.” Based on prior literature, we hypothesize that SCC will be higher for African Americans than for Caucasians. We predict that SCC will be associated with attrition for both groups, but will have a stronger association among African Americans because prior literature shows that stereotype confirmation concerns have the strongest negative impact on African Americans [13]. Variables associated with attrition in prior studies, including demographic factors and pre-treatment symptom severity, are also examined.

## Methods

### Participants

Participants were 74 individuals diagnosed with social anxiety disorder who received treatment as part of a larger randomized controlled trial [17] comparing Virtual Reality Exposure Therapy (VRE) [18], Exposure Group Therapy (EGT) [19], and a wait-list control group. Approval from the Georgia State University Institutional Review Board (IRB) was obtained for this study and each participant provided informed consent prior to participating. Participants were included in the current study if they were literate in English and had a primary diagnosis of social anxiety disorder with a primary fear of public speaking, as determined by the Structured Clinical Interview for the Diagnostic and Statistical Manual of Mental Disorders, Fourth Edition (SCID-IV) [20]. Exclusion criteria included history of seizure disorder, mania, schizophrenia, or other psychoses, as well as prominent suicidal ideation, or current alcohol or drug abuse or dependence. For the present study, only individuals who self-identified as “African American” (42%, *n* = 31) or “Caucasian” (58%, *n* = 43) were included.

The sample had slightly more females (61%, *n* = 45) than males, with an average age of *M* = 39.56, *SD* = 11.43. The sample was well educated, 33% completed college, and 44% reported their relationship status was married. Most were middle class, with 43% having an annual income of $50,000 or more. There were 42 participants (57%) in the group treatment (EGT) and 32 participants (43%) in the virtual reality treatment (VRE). The majority of the participants did not have a comorbid diagnosis (*n* = 61, 81%).

### Measures

#### Social anxiety

The Liebowitz Social Anxiety Scale-Self Report (LSAS-SR) [21] is a self-report version of a clinician-administered measure of social anxiety [22] consisting of 24 items using a 4-point rating scale, with a total score that ranges from 0–144. Higher scores indicate greater social anxiety. The self-report measure performs similarly to the clinician-administered version [21], with reliability estimates that range from .88 to .95 [23]. Reliability for the current study was excellent with α = 0.94.

#### Stereotype confirmation concerns

The stereotype confirmation concerns scale (SCCS) is an 11-item measure of participants’ fears that they are confirming a stereotype about one’s social group over a range of social and behavioral domains [13]. Items are rated on a 7-point Likert type scale ranging from 1 (*never*) to 7 (*always*) and a total score ranging from 11 to 77, with higher scores reflecting greater concern over confirming stereotypes. Participants rate how often over the past three months they have been “concerned that by ___ [they] might appear to be confirming a stereotype about [their] group”. Examples of items include “dressing a certain way”, “talking in a certain way”, and “revealing your socioeconomic status”. The reference group (e.g., age, gender, race) for which respondents rate stereotype confirmation concerns is not specified in this measure. Internal consistency for the SCCS is excellent (Chronbach’s α = .91) [13]. Reliability for the current study was excellent with α = 0.92.

### Procedures

#### Screening

Potential participants were first screened by phone and then invited for an in-person assessment, during which a doctoral candidate administered the Structured Clinical Interview for the DSM-IV (SCID) to determine if the individual met criteria for a primary diagnosis of social anxiety disorder and other comorbid disorders. Four doctoral candidates in clinical psychology, supervised by a licensed psychologist, conducted all assessment procedures. Inter-rater reliability was calculated for a randomly selected subset (10%) of interviews. Agreement on the primary diagnosis was 100%, with one disagreement on illness severity.

#### Treatment

Both treatments consisted of eight sessions of cognitive behavioral therapy designed to target processes identified in the psychopathology literature as maintaining social anxiety disorder, including self-focused attention, negative perceptions of self and others, perceptions of lack of emotional control, and realistic goal setting for social situations. Both treatments were administered according to a manual [18,24]. The primary difference between the two therapies was the method of exposure. During exposure group therapy, exposure was delivered using other group members; during virtual reality treatment, exposure was delivered using virtual reality. The original developers of the treatments manuals provided ratings of adherence to protocols for a randomly selected subset of videotaped sessions (14%). Compliance to the treatment protocols was quite good for each treatment, with 92% and 93% of the essential elements of the protocols being completed for VRE and EGT, respectively, and one infraction for each treatment type across all sessions reviewed. See Anderson et al., [17] for study details.

### Data analysis

A hierarchical logistic regression [25] was used to identify predictors of dropout status in each treatment condition. Dropout status was defined as a dichotomous variable such that 0 = treatment completer and 1 = dropout. Participants in the EGT condition were classified as a dropout if they missed more than two EGT sessions, whereas those in the VRE condition were considered a dropout if they missed more than one session. Dropouts were conceptualized differently for each condition because of challenges with scheduling. EGT sessions could not be rescheduled based on the needs of a single participant, whereas VRE sessions could be altered to accommodate the needs of a specific participant. Such difficulties have been discussed in prior studies [26].

A hierarchical logistic regression was used to identify predictors of dropout from treatment. The order of entry for the variables was based on their support from previous literature; those that were found to be significant predictors in previous literature were entered first and exploratory variables were entered in the final step. Treatment type (VRE or EGT) was first entered as a covariate in order to account for the differences in therapy format. Significant predictors from prior research (i.e., gender, age, and pretreatment severity) were included in the second step of the model. Finally, racial group, stereotype confirmation concerns, and their interaction were included. Given the exploratory nature of the last step, variables in the final step were entered in a stepwise fashion with backwards removal to identify an optimally fitting model.

## Results

Descriptive information is provided in Table 1. Consistent with our hypothesis, there was a significant difference in stereotype confirmation concern scores between African Americans and Caucasians, *F* (1, 76) = 9.74, *p* < 0.01. African Americans (*M* = 35.50, *SD* = 17.46) reported significantly higher scores than Caucasians (*M* = 24.63, *SD* = 13.29).

**Table 1:** **Descriptive statistics for treatment completers and treatment dropouts**

	**Completer**		**Dropout**	
	**VRE (n = 27)**	**EGT (** ***n*** **= 27)**	**VRE (** ***n*** **= 5)**	**EGT (** ***n*** **= 15)**
	*M (SD)*	*M (SD)*	*M (SD)*	*M (SD)*
LSAS-SR	51.04 (22.07)	51.03 (20.80)	63.80 (18.71)	56.04 (16.30)
SCCS	26.96 (14.54)	25.63 (14.28)	30.60 (9.91)	39.67 (2.69)
Age	38.74 (11.53)	43.30 (11.84)	37.60 (13.05)	34.27 (8.22)
	*n* (%)	*n* (%)	*n* (%)	*n* (%)
African American	9 (33%)	9 (33%)	1 (20%)	12 (80%)
Male	8 (30%)	14 (52%)	1 (20%)	6 (40%)
College Graduates	18 (67%)	20 (74%)	3 (60%)	9 (60%)
Unpartnered	9 (33%)	12 (44%)	3 (60%)	12 (80%)

Note. VRE = Virtual Reality Exposure Therapy. EGT = Exposure Group Therapy. LSAS-SR = Liebowitz Social Anxiety Scale. SCCS = Stereotype Confirmation Concerns Scale.

A hierarchical logistic regression was used to determine predictors of dropout from treatment. Hosmer and Lemeshow goodness of fit tests suggested that the model demonstrated adequate fit in the first step (**χ**^2^ = 5.47, *p* = 0.71). There were no indicators of multicollinearity (the Tolerance value range from .84 to.94 and the VIF values range from 1.07 to 1.37). The normality of the variables was also examined; all of the variables were normally distributed, with low skew and kurtosis. The assumption of linearity of the logit was also met (the logistic regression was run with the interaction terms of each continuous predictor and its log, none of which were significant predictors). As shown in Table 2, in the second step group treatment was associated with an increased likelihood for dropout (OR = 3.77, *p* = 0.04, 95% CI [1.10-12.98]), but age, gender, and pretreatment severity were not significant predictors of attrition. Racial group, stereotype confirmation concerns, and their interaction were entered in the final step and, given the exploratory nature of these variables, stepwise entry with backwards removal was used for the final step. Hosmer and Lemeshow goodness of fit tests suggested that the final model demonstrated good fit (**χ**^2^ = 6.67, *p* = 0.57); in addition Nagelkerke *R*^*2*^ = 0.28 and -*2LL* = 70.44. As shown in Table 2, the final model showed that stereotype confirmation concerns (B = 0.03, OR = 1.04, *p* = 0.03, 95% CI [1.01-1.08] and participating in group therapy (B = 1.35, OR = 3.84, *p* = 0.04, 95% CI [1.04, 14.13] were associated with an increased likelihood for dropout. There was not a significant effect of racial group or its interaction with stereotype confirmation concerns, and their removal from the model did not decrease model fit.

**Table 2:** **Logistic regression identifying indicators of attrition**

	**B**	**SE**	**OR**	**95% CI**
*Step 1: Treatment as Covariate*				
Treatment Type (Group)	1.10	0.58	3.00	0.96-9.42
*Step 2: Inclusion of Previously Supported Predictors*				
Treatment Type (Group)	1.33	0.63	3.77*	1.01-12.98
Age	−0.06	0.03	0.95	0.89-1.00
Gender (Male)	−0.17	0.62	0.85	0.25-2.83
Pretreatment Severity	0.02	0.01	1.02	0.99-1.05
*Step 3: Final Model (Backwards Deletion of SCCS, Race, & their interaction)*				
Treatment Type (Group)	1.35	0.66	3.84*	1.043-14.125
Age	−0.06	0.03	0.95	0.893-1.001
Gender (Male)	−0.27	0.66	0.76	0.211-2.759
Pretreatment Severity	0.01	0.02	1.01	0.980-1.042
SCCS	0.04	0.02	1.04*	1.005-1.082

Note: * = *p* < 0.05. OR = Odds Ratio. 95% CI = 95% Confident Interval. SCCS = Stereotype Confirmation Concerns Scale.

## Discussion

As hypothesized, stereotype confirmation concerns were related to dropout from cognitive behavioral therapy for social anxiety disorder. Given that fear of negative evaluation is a primary component of social anxiety [27], concerns about negative evaluation of one’s social group and oneself as a representative of that group may exacerbate fear of social situations and lead to avoidance (dropout). For example, research on meta-stereotypes (individuals’ perceptions of others’ stereotypes about their own social group) finds that Blacks believe Whites endorse negative racial stereotypes about their group and view them as violent, unintelligent, and lazy [28]. Clinically, these findings have several implications. The results suggest that therapists should be aware of the impact of stereotype confirmation concerns to potentially prevent attrition. A multicultural perspective can be incorporated into various therapies and culturally-adapted interventions have been found to have a moderately strong benefit [29]. In keeping with multicultural competency, it may be beneficial for clinicians to measure clients’ stereotype confirmation concerns before treatment, as such fears may also be related to client’s anticipated negative outcomes and clients’ avoidant behavior, including premature dropout from therapy. Gaining information on clients’ social identities and fears of confirming stereotypes would allow for consideration of their influence on the clients’ feared social situations, which could enrich exposure.

Participants assigned to group therapy were more likely to dropout than those who assigned to virtual reality therapy delivered in an individualized format. Group therapy may be more difficult for those with social anxiety to complete because in vivo social interactions of any kind may evoke greater anxiety and induce avoidance [17]. Although this study was not sufficiently powered to look at the different treatment conditions, it may be the case that stereotype confirmation concerns are more relevant for group treatment than for individual treatment. The presence of individuals from multiple social groups in group therapy may make stereotypes more salient and therefore induce stereotype confirmation concerns, which in turn could increase the likelihood for dropout. It may be useful to consider social identities and fear of confirming negative stereotypes when discussing interpersonal patterns or anxiety evoked within the group setting. This is an area for future research.

The findings of the current study should be considered within the context of its limitations. These data were collected as part of an efficacy-focused RCT in a psychology training clinic. Although participants were recruited from the community, the findings may not be generalizable to effectiveness settings, and future research is needed to evaluate the impact of stereotype confirmation concerns on attrition in other care settings. Importantly, the measure of stereotype confirmation concerns did not specifically assess which stereotype(s) participants were concerned about confirming (e.g., gender, race, religion). The stereotypes that drive an individual’s uncertainty and apprehension can vary widely, because each person identifies with multiple social groups (e.g., gender, race, religion), each of which may have stereotypes (both negative and positive) that people view themselves as being at risk of perpetuating. Future research on stereotype confirmation concerns and social anxiety would benefit from gathering qualitative information on the nature of the stereotypes that participants fear they will reinforce. Finally, due to sample size limitations, the current study only included those who self-identified as African American or Caucasian. Therefore, the extent to which these associations can be applied to other racial and ethnic groups is limited.

## Conclusions

The current study provides new insights into the issue of attrition in social anxiety treatments and suggests that future research could benefit from focusing on predictors of attrition that address one’s social identities within the context of cognitive behavioral therapy.
